# Supplementary material for: Netcombin: An algorithm for constructing optimal phylogenetic network from rooted triplets
Source: PLoS One. 2020 Sep 18;15(9):e0227842. doi: 10.1371/journal.pone.0227842 (PMC7500971; doi:10.1371/journal.pone.0227842)
Supplement: S4 File — (ZIP) [file pone.0227842.s004.zip › SequenceData-Triplet/sequenceData-10-20-30-0-Triplet/length 300, numseq 30, rate 10^-7/5 seq.pdf]

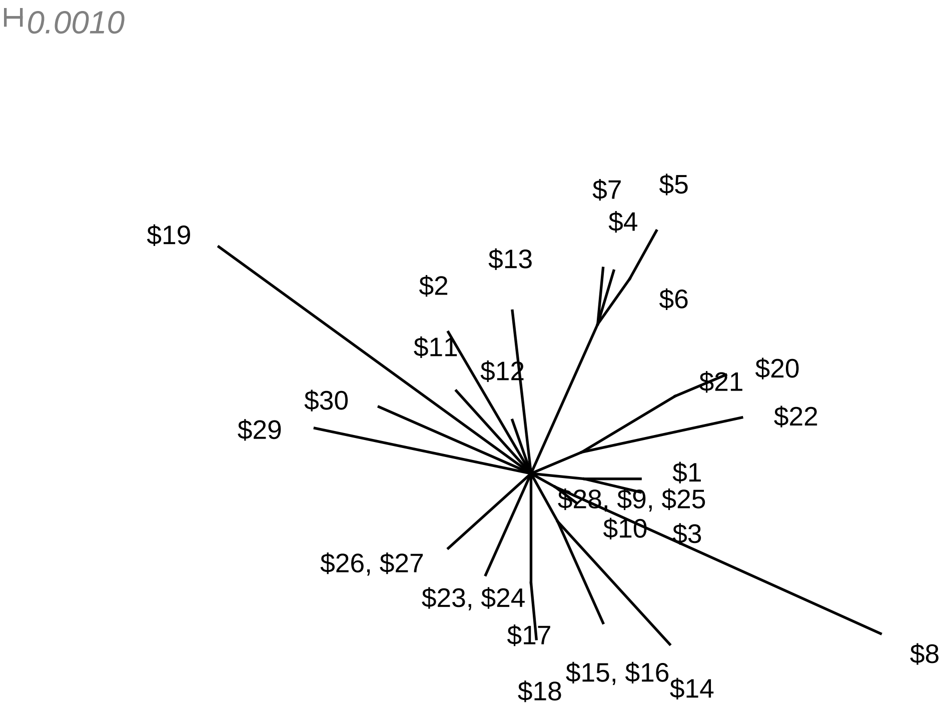
0.0010
$7
$5
$4
$19
$13
$2
$6
$11
$12
$20
$21
$30
$22
$29
$1
$28, $9, $25
$10
$3
$26, $27
$23, $24
$17
$8
$15, $16
$14
$18
